# *EVI1* Promotes the Proliferation and Invasive Properties of Human Head and Neck Squamous Cell Carcinoma Cells

**DOI:** 10.3390/ijms23031050

**Published:** 2022-01-19

**Authors:** Alexander Michael Grandits, Sophie Bromberger, Gerwin Heller, Barbara Andrea Reinoehl, Erwin Tomasich, Klaudia Schossleitner, Anna Sophie Berghoff, Thorsten Fuereder, Rotraud Wieser

**Affiliations:** 1 Division of Oncology, Department of Medicine I, Medical University of Vienna, Waehringer Guertel 18-20, 1090 Vienna, Austria; alexander.grandits@meduniwien.ac.at (A.M.G.); gerwin.heller@meduniwien.ac.at (G.H.); barbara.andi.reinoehl@gmail.com (B.A.R.); erwin.tomasich@meduniwien.ac.at (E.T.); anna.berghoff@meduniwien.ac.at (A.S.B.); thorsten.fuereder@meduniwien.ac.at (T.F.); 2 Comprehensive Cancer Center, Spitalgasse 23, 1090 Vienna, Austria; 3 Skin and Endothelium Research Division, Department of Dermatology, Medical University of Vienna, Waehringer Guertel 18-20, 1090 Vienna, Austria; sophie.bromberger@meduniwien.ac.at (S.B.); klaudia.schossleitner@meduniwien.ac.at (K.S.)

**Keywords:** squamous cell carcinoma of head and neck, EVI1, MECOM, PRDM3, cell proliferation, lymphatic metastasis, neoplasm metastasis, gene expression regulation, molecular targeted therapy

## Abstract

Head and neck squamous cell carcinoma (HNSCC) is a frequent malignancy with a poor prognosis. So far, the EGFR inhibitor cetuximab is the only approved targeted therapy. A deeper understanding of the molecular and genetic basis of HNSCC is needed to identify additional targets for rationally designed, personalized therapeutics. The transcription factor EVI1, the major product of the *MECOM* locus, is an oncoprotein with roles in both hematological and solid tumors. In HNSCC, high EVI1 expression was associated with an increased propensity to form lymph node metastases, but its effects in this tumor entity have not yet been determined experimentally. We therefore overexpressed or knocked down *EVI1* in several HNSCC cell lines and determined the impact of these manipulations on parameters relevant to tumor growth and invasiveness, and on gene expression patterns. Our results revealed that *EVI1* promoted the proliferation and migration of HNSCC cells. Furthermore, it augmented tumor spheroid formation and the ability of tumor spheroids to displace an endothelial cell layer. Finally, *EVI1* altered the expression of numerous genes in HNSCC cells, which were enriched for Gene Ontology terms related to its cellular functions. In summary, *EVI1* represents a novel oncogene in HNSCC that contributes to cellular proliferation and invasiveness.

## 1. Introduction

Head and neck squamous cell carcinoma (HNSCC) is the sixth most common malignancy worldwide, with more than 830,000 new diagnoses and more than 430,000 deaths per year [1,2]. Primary tumors are predominately located in the oral cavity, oropharynx, hypopharynx, or larynx [2]. Excessive consumption of alcohol and/or tobacco products are the primary risk factors and are associated with 75% of cases [1,2]. More recently, human papilloma virus (HPV) infection has been identified as an additional etiological factor with increasing importance, particularly in younger men, and is associated with better outcomes [1,2]. HNSCC is a genetically and molecularly heterogeneous disease. Among the most important genetic lesions are *TP53* mutations and mutations in the pRb pathway, both of which represent early events [1]. Moreover, overexpression of the epidermal growth factor receptor (EGFR) is observed in 80–90% of HNSCC cases and indicates a poor prognosis [1,2].

While early-stage HNSCC, even if HPV-negative, has a 5-year overall survival (OS) rate of 70–90%, the majority (two-thirds) of patients present with advanced disease, which is associated with a 5-year OS of only around 25–50%, depending on the primary tumor site [2]. Treatment is multimodal, comprising surgery, radiation, and platinum compound-based chemotherapy [1,2]. So far, the EGFR inhibitor cetuximab, a monoclonal antibody that competitively inhibits ligand binding, is the only approved targeted treatment, but its efficacy is limited and resistance is frequent [1,2]. Most recently, immune checkpoint inhibitors were also successfully established for the treatment of recurrent/metastatic HNSCC [1,2]. Overall, however, treatment success is limited, relapses are frequent, and survival outcomes are unsatisfactory [1,2].

Ecotropic viral integration site 1 (EVI1), one of several transcript and protein variants derived from the *MDS1-EVI1* complex (*MECOM*, alias *PRDM3*) locus in chromosome band 3q26 [3,4], was first discovered because its transcriptional activation by retroviral insertion promoted myeloid leukemia in mice [5]. EVI1 is a transcription factor with two zinc finger domains, comprising seven and three zinc finger motifs, respectively [3,4]. It has been shown to bind to DNA in a sequence-specific manner, to interact both with other sequence-specific transcription factors and with transcriptional cofactors, and to regulate numerous target genes [3,4,6,7,8,9,10,11,12]. Targeted disruption of the *Mecom* locus in mice revealed multiple roles in embryonic development [13]. Moreover, the key roles of this gene in hematopoietic stem cells have been described [14,15]. Beyond this, *MECOM*, and particularly its product EVI1, acts as an oncogene in a variety of different tumor entities, as revealed both by investigations on corresponding patient cohorts and by experimental studies. A large body of work has focused on the role of *EVI1* in myeloid malignancies, particularly in acute myeloid leukemia (AML). Overexpression of *EVI1* is present in around 10% of patients with AML and is associated with a dismal prognosis [16,17,18]. Moreover, *EVI1* promoted the key properties of AML stem cells [19]. Increasingly, the role of *EVI1* in solid tumors is also becoming recognized. Thus, EVI1 was overexpressed as compared with corresponding healthy tissues, associated with parameters reflecting disease aggressiveness, and/or correlated with shorter survival in a variety of tumor entities, including glioblastoma multiforme [20], triple negative breast cancer [21], clear cell renal cell carcinoma [22], lung squamous cell carcinoma [23], cholangiocarcinoma [24,25], prostate cancer [26], serous epithelial ovarian cancer [27], pancreatic ductal adenocarcinoma [28], and nasopharyngeal carcinoma [9]. In corresponding experimental models, *EVI1* promoted proliferation, colony formation, anchorage-independent growth, migration, invasion, tumor sphere formation (an in vitro surrogate for cancer stem cell activity), epithelial-to-mesenchymal transition, resistance to drug-induced apoptosis, and/or tumor growth and metastasis formation in syngeneic or xenotransplantation mouse models [8,9,11,12,25,26,27,28,29,30]. The numerous molecular mechanisms implicated in these biological functions include inhibition of TGF-β signaling [27] and activation of KRAS [28], AKT [25,29], mTOR [8], and Wnt signaling [9,11].

In HNSCC, increased expression of EVI1 in primary tumors correlated with the presence of lymph node metastases [31], which are usually associated with an advanced disease stage and worse prognosis. However, to the best of our knowledge, the functional effects of *EVI1* in this tumor entity have not yet been described. Here, we report that *EVI1* promoted proliferation and properties related to tumor invasiveness, and regulated the expression of numerous genes in HNSCC cells.

## 2. Results

### 2.1. Establishment of HNSCC Cell Lines with Experimentally Altered EVI1 Expression

**Figure 1:**
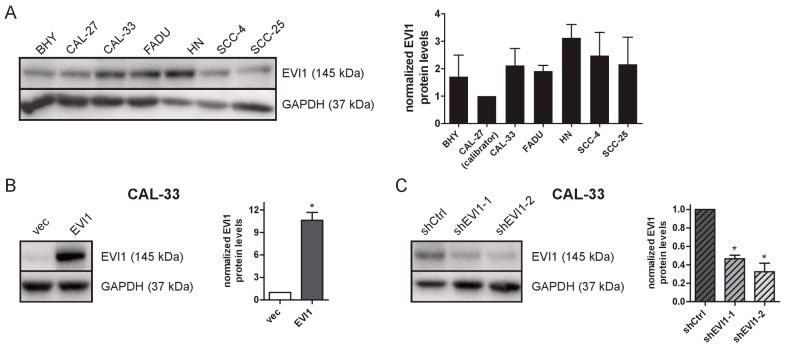
Expression of EVI1 in native and transduced HNSCC cell lines. (**A**) EVI1 protein levels in seven HNSCC cell lines were determined by immunoblot analysis; GAPDH was used as a loading control. Left panel, representative immunoblot; right panel, quantification. EVI1 levels were normalized to GAPDH levels and to CAL-27 cells. Means + SEM, *n* = 3. (**B**) CAL-33 cells were transduced with a retroviral vector containing the human *EVI1* cDNA (EVI1) or with an empty vector as a control (vec). Overexpression of EVI1 was confirmed by immunoblot analysis. (**C**) CAL-33 cells were transduced with lentiviral vectors containing shRNAs targeting human *EVI1* (shEVI1-1, shEVI1-2) or a control shRNA targeting the *renilla luciferase* gene (shCtrl). Knock-down of EVI1 was confirmed by immunoblot analysis. (**B**,**C**) Left panels, representative immunoblots; right panels, quantification using GAPDH for normalization. Means + SEM, *n* = 3. * *p* < 0.05; one-sample *t*-test (**B**) or one-sample *t*-test with Bonferroni correction for multiple testing (**C**).

High EVI1 protein expression was associated with an increased propensity to form lymph node metastases in HNSCC [31]. To investigate the biological functions of *EVI1* in this tumor entity, we first determined its expression in a panel of human HNSCC cell lines. BHY, CAL-27, CAL-33, FADU, HN, SCC-4, and SCC-25 all expressed moderate and somewhat variable levels of EVI1 mRNA and protein (Figure 1A and Figure S1A,B). Further experiments focused on cell lines derived from carcinomas of the oral cavity because of the frequency and aggressive nature of this HNSCC sub-entity [2]. CAL-33, CAL-27, SCC-25, and SCC-4 cells were transduced with a retroviral *EVI1* expression vector or with an empty vector as a control. Transduced cells were sorted by flow cytometry for vector-encoded enhanced green fluorescent protein (eGFP), and overexpression of EVI1 was confirmed by immunoblot analysis (Figure 1B and Figure S1C–E). The resulting *EVI1* overexpression and empty vector control cell lines are designated by appending “_EVI1” and “_vec”, respectively, to the respective cell line names (e.g., CAL-33_EVI1, CAL-33_vec). Moreover, CAL-33 cells were infected with lentiviral vectors containing one of two different shRNAs targeting *EVI1* (shEVI1-1, shEVI1-2) or a control shRNA (shCtrl). After sorting for the vector-encoded fluorescent marker Venus, successful knock-down of EVI1 in the resulting cell lines CAL-33_shEVI1-1 and CAL-33_shEVI1-2 vs. CAL-33_shCtrl was corroborated by immunoblot analysis (Figure 1C).

### 2.2. EVI1 Promotes the Proliferation of HNSCC Cells

**Figure 2:**
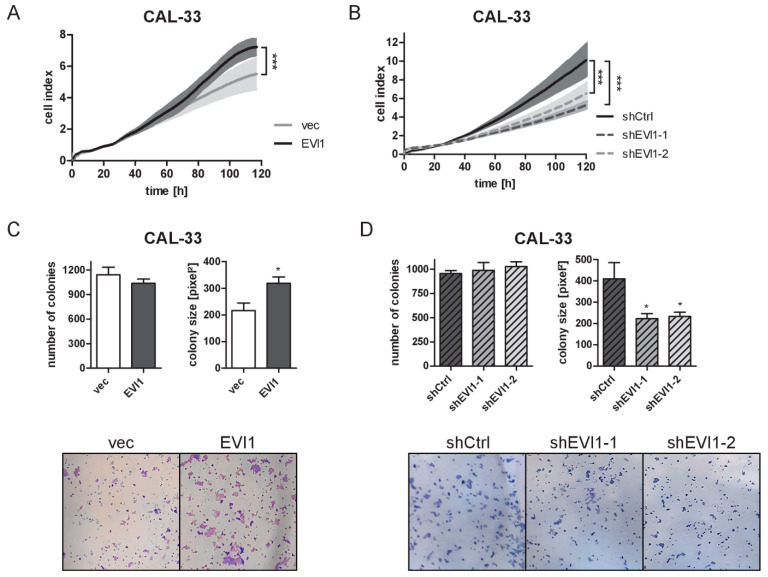
EVI1 promotes the proliferation of CAL-33 cells. (**A**,**B**) Proliferation of CAL-33_EVI1 and CAL-33_vec cells (**A**), or of CAL-33_shEVI1-1, CAL-33_shEVI1-2, and CAL-33_shCtrl cells (**B**) was monitored in real-time for 120 h. Impedance values determined by the xCELLigence system were normalized to the 24 h time point. Means ± SEM, *n* = 3. *** *p* < 0.001, according to the *anova.lme* function of the R package *nlme*. (**C**,**D**) Colony formation. CAL-33_EVI1 and CAL-33_vec cells (**C**), or CAL-33_shEVI1-1, CAL-33_shEVI1-2, and CAL-33_shCtrl cells (**D**) were seeded at low densities into six-well-plates and stained with trypan blue after 11 days. Top panels: quantification of colony numbers and sizes; bottom panels: representative well areas. Means + SEM, *n* = 3. * *p* < 0.05; Student’s two-sided *t*-test (**C**) or one-way ANOVA followed by Dunnett’s multiple comparison test (**D**).

To evaluate whether *EVI1* affected the proliferation of HNSCC cells, increases in cell numbers were monitored in real-time by measuring impedance changes in cell culture plates with integrated electrodes. These experiments showed that overexpression of *EVI1* promoted cellular proliferation and knock-down of *EVI1* decreased it (Figure 2A,B and Figure S2A–C). Moreover, when cells were seeded into standard culture dishes at a low density, experimental manipulation of *EVI1* expression, albeit without an impact on the number of colonies formed, affected colony size in a manner consistent with a growth-promoting effect of *EVI1* (Figure 2C,D and Figure S2D–F). The positive impact of *EVI1* on cell proliferation was observed in all five investigated cell line models (one knock-down and four overexpression models) for both assay types.

### 2.3. EVI1 Promotes Spheroid Formation by HNSCC Cells

**Figure 3:**
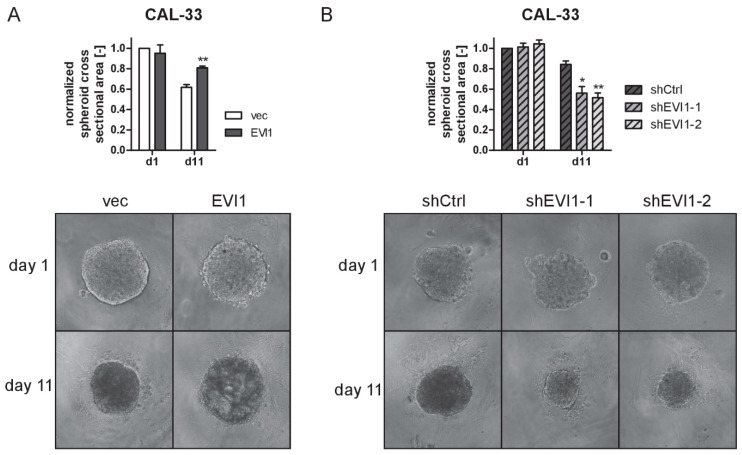
EVI1 increases the size of CAL-33 spheroids. Tumor spheroids from CAL-33_EVI1 and CAL-33_vec cells (**A**), or from CAL-33_shEVI1-1, CAL-33_shEVI1-2, and CAL-33_shCtrl cells (**B**) were allowed to form over a period of 11 days. Top panels: quantification of spheroid sizes; bottom panels: representative spheroids. Means + SEM, *n* = 3. *, *p* < 0.05, ** *p* < 0.01; Student’s two-sided *t*-test (**A**) or one-way ANOVA followed by Dunnett’s multiple comparison test (**B**).

The ability to form spheroids is thought to reflect the stem cell-related properties of cancer cells [32]. *EVI1* has previously been implicated in the activities of both normal and malignant stem cells [9,14,19,26]. To query whether *EVI1* affected spheroid formation in HNSCC cells, cell lines with experimentally manipulated *EVI1* expression were seeded into ultra-low attachment plates, and the sizes of the spheroids generated under these conditions were monitored over several days. CAL-33 cells formed the most compact and stable spheroids, and overexpression and knock-down of *EVI1* increased and decreased, respectively, spheroid size after 11 days (Figure 3A,B). Spheroids derived from CAL-27, SCC-25, and SCC-4 cells started to disintegrate much earlier, and therefore were monitored for only 3 days. After this time, *EVI1* overexpression had significantly increased the size of CAL-27 spheroids, but had no effect on SCC-25- and SCC-4-derived spheroids (Figure S3A–C). Thus, although its impact may be less readily observed in cell lines with a reduced basal ability to generate spheroids, *EVI1* promoted the formation of tumor spheroids in HNSCC cell lines.

### 2.4. EVI1 Promotes the Migration and Invasive Properties of HNSCC Cells

Because analyses of primary HNSCC patient samples had suggested a possible role of *EVI1* in the formation of lymph node metastases [31], we next asked whether manipulation of *EVI1* expression would affect the migration and invasiveness of HNSCC cell lines as well as endothelial barrier disruption by HNSCC cell spheroids.

**Figure 4:**
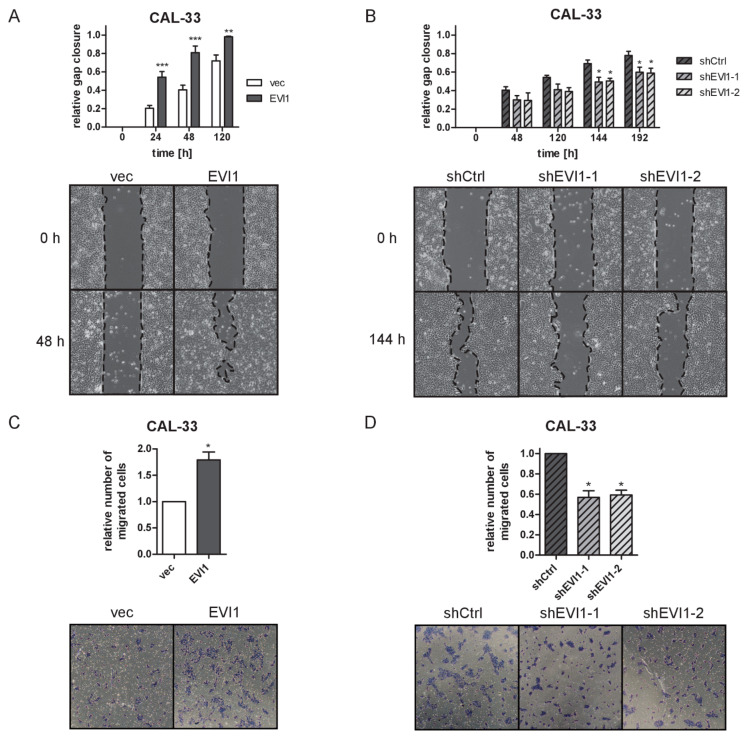
EVI1 enhances the migration of CAL-33 cells. (**A**,**B**) Scratch assay. CAL-33_EVI1 and CAL-33_vec cells (**A**), or CAL-33_shEVI1-1, CAL-33_shEVI1-2, and CAL-33_shCtrl cells (**B**) were grown to 90% confluence in a regular growth medium. The medium was changed to low serum conditions (0.2% fetal bovine serum (FBS)), scratches were introduced 24 h later, and gap closure was monitored at the indicated time points thereafter. Top panels: quantification; bottom panels: representative experiments. Means + SEM, *n* = 3. * *p* < 0.05, ** *p* < 0.01, *** *p* < 0.001, two-way ANOVA followed by Bonferroni’s post-hoc test. (**C**,**D**) Transwell migration assay. Serum-starved CAL-33_EVI1 and CAL-33_vec cells (**C**), or CAL-33_shEVI1-1, CAL-33_shEVI1-2, and CAL-33_shCtrl cells (**D**) were seeded into transwell inserts and allowed to migrate towards a medium containing 10% FBS. After 24 h, cells at the bottom of the transwell membranes were stained with trypan blue. Top panels: quantification; bottom panels: images from representative experiments. Means + SEM, *n* = 3. * *p* < 0.05; one-sample *t*-test (**C**) or one-sample *t*-test with Bonferroni correction for multiple testing (**D**).

Indeed, overexpression and knock-down of *EVI1* in CAL-33 cells accelerated and retarded, respectively, gap closure in a scratch assay (Figure S4A,B). To rule out the possibility that this effect was secondary to the impact of *EVI1* on cell proliferation, the assay was repeated in media containing 0.2% fetal bovine serum (FBS), which effectively impeded the proliferation of CAL-33 cells (Figure S4C). Gap closure generally took longer under these conditions, yet the effects of *EVI1* overexpression and knock-down were still clearly evident (Figure 4A,B). Similarly, *EVI1* overexpression promoted the migration of CAL-27 and SCC-25 cells under proliferation-minimizing reduced serum conditions (Figure S4D–G). The assay could not be performed for SCC-4 cells because their tight adherence to each other and/or the surface of the culture dish precluded the introduction of an evaluable scratch into the cell layer. The results of the scratch assays were corroborated by transwell migration assays: *EVI1* overexpression promoted the migration of CAL-33, CAL-27, and SCC-4 cells from serum-free to serum-containing media, and its knock-down in CAL-33 cells inhibited migration under these conditions (Figure 4C,D and Figure S5A–C).

To assess the effect of *EVI1* on cell invasiveness, transwell assays in which the upper wells were coated with Matrigel^®^ were conducted. Overexpression of *EVI1* enhanced the invasiveness of CAL-33, CAL-27, and SCC-4 cells (Figure S5D–H).

**Figure 5:**
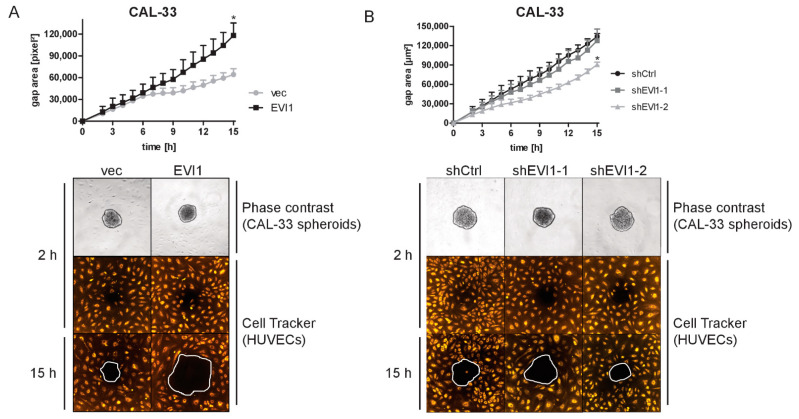
EVI1 enhances the displacement of endothelial cells by CAL-33-derived spheroids. (**A**,**B**) Spheroids were generated from CAL-33_EVI1 and CAL-33_vec cells (**A**), or from CAL-33_shEVI1-1, CAL-33_shEVI1-2, and CAL-33_shCtrl cells (**B**). They were placed onto confluent HUVECs that had been stained with Cell Tracker Dye, and HUVEC displacement was followed by live-cell imaging. Top panels: quantification; bottom panels: images of representative experiments. Means + SEM, *n* = 3. Differences in gap areas were tested for statistical significance after 15 h. * *p* < 0.05; Student’s two-sided *t*-test (**A**) or one-way ANOVA followed by Dunnett’s multiple comparison test (**B**).

Finally, the ability of tumor spheroids to disrupt an endothelial cell monolayer was tested. One- to 2-day-old spheroids formed by CAL-33, CAL-27, and SCC-25 derivative cell lines (i.e., spheroids whose size was not yet affected by *EVI1*) were placed onto a monolayer of human umbilical vein endothelial cells (HUVECs). The ability of the spheroids to displace endothelial cells was monitored over time. After 15 h, CAL-33_EVI1- and SCC-25_EVI1-derived spheroids had formed significantly larger gaps in the HUVEC monolayer than their empty vector-containing counterparts. Knock-down of *EVI1* in CAL-33 cells had the opposite effect (Figure 5A,B and Figure S6A,B). SCC-4 spheroids were too fragile to be used in this assay.

In summary, *EVI1* promoted the properties of HNSCC cell lines that are related to tumor migration and invasion.

### 2.5. EVI1 Regulates the Expression of Genes Implicated in Epithelial Development, Adhesion, and Proliferation in HNSCC Cells

**Figure 6:**
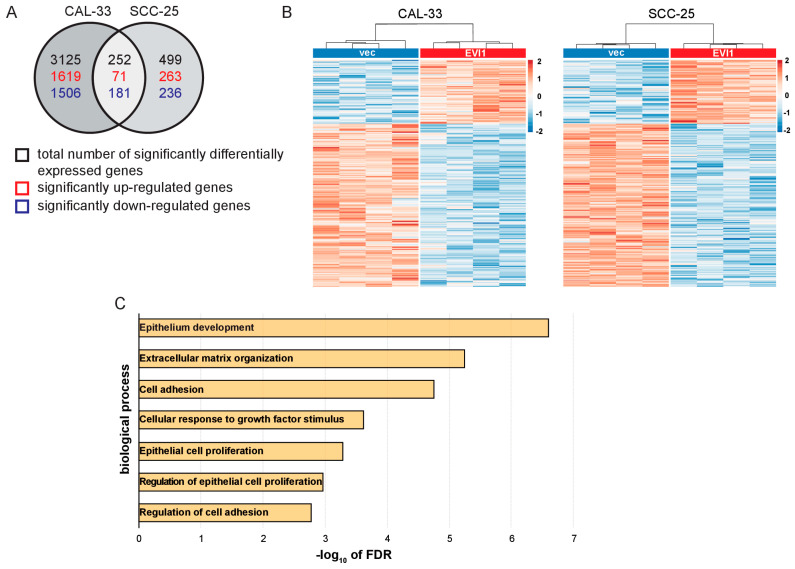
EVI1 regulates genes implicated in epithelial development, adhesion, and proliferation in HNSCC cells. Genome-wide gene expression patterns in CAL-33_EVI1, CAL-33_vec, SCC-25_EVI1, and SCC-25_vec cells were determined by RNA-seq. (**A**) Venn diagram showing the numbers of genes differentially expressed at a false discovery rate (FDR) of <0.1 upon experimental expression of EVI1 in CAL-33 and/or SCC-25 cells. (**B**) Heatmap of 252 genes consistently regulated by EVI1 in CAL-33 and SCC-25 cells. The rows and columns represent genes and biological replicates, respectively. Blue, low expression; red, high expression. (**C**) Selected categories from the Gene Ontology enrichment analysis of the 252 commonly deregulated genes, performed using ShinyGO (v0.61).

EVI1 exerts its biological functions primarily by acting as a transcription factor [3,4,6,7,8,9,10,11,12]. We therefore aimed to identify putative EVI1 target genes in HNSCC cells. RNA was extracted from quadruplicate cultures of CAL-33_vec, CAL-33_EVI1, SCC-25_vec, and SCC-25_EVI1 cells and subjected to RNA sequencing (RNA-seq). In CAL-33 cells, 1690 and 1687 genes were up- and downregulated, respectively, at a false discovery rate (FDR) of <0.1 upon experimental expression of *EVI1* (Figure 6A, Supplementary Table S1A). In SCC-25 cells, 334 significantly up- and 417 significantly downregulated genes were identified (Figure 6A, Supplementary Table S1B); 71 and 181 genes were up- and downregulated, respectively, in response to *EVI1* expression in both cell lines (Figure 6A,B; Supplementary Table S1C). Gene Ontology analysis revealed, among others, the terms epithelium development, extracellular matrix organization, cell adhesion, response to growth factor, and epithelial cell proliferation to be significantly (FDR < 0.1) enriched among the 252 genes that were regulated by EVI1 in a consistent manner in both cell lines (Figure 6C, Supplementary Table S1D). To probe the relevance of the identified EVI1-dependent gene expression signature for primary human HNSCC, the TCGA HNSCC dataset (Firehose Legacy, *n* = 522 with RNA-seq data available) was used. Transcript levels of 15 of the 71 genes (21%) that were upregulated by EVI1 in both cell lines correlated positively (Spearman ρ > 0.3) with *EVI1* mRNA levels in the primary samples. Of the 181 genes downregulated by EVI1 in both cell lines, 24 (13%) correlated negatively (Spearman ρ < −0.3) with *EVI1* in the TCGA data (Supplementary Table S1E). Taken together, these results show that EVI1 regulates numerous genes in HNSCC cells, and that these genes are significantly enriched for processes relevant to its biological functions in these cells. 

## 3. Discussion

Analyses of patient samples as well as functional studies using in vitro and in vivo model systems have implicated EVI1, the major protein product of the *MECOM* locus, as an oncoprotein in myeloid leukemias and in various solid tumor entities [8,9,11,12,14,16,17,18,19,20,21,22,23,24,25,26,27,28,29,30,33,34]. In HNSCC, high EVI1 expression was associated with an increased probability of the presence of lymph node metastases [31], but, to the best of our knowledge, no characterization of the functional roles of this gene in this tumor entity has been reported so far. In the studies described here, we used three different HNSCC cell lines to overexpress *EVI1* and a fourth to perform both overexpression and knock-down experiments. Functional assays with the resulting derivative cell lines demonstrated that *EVI1* promoted the proliferation of HNSCC cells and augmented the size of tumor spheroids formed by them. Furthermore, *EVI1* enhanced the migration and invasiveness of HNSCC cell lines, as well as the ability of spheroids derived from them to displace endothelial cells (under conditions where the impact of *EVI1* on spheroid size was not yet manifest). These properties are relevant to the initiation of metastasis formation and thus are in agreement with the correlation between EVI1 expression and the formation of lymph node metastases observed in patient samples [31]. The same study did not find any prognostic significance of EVI1 expression in HNSCC [31]; accordingly, we found only marginal effects of *EVI1* on cellular sensitivity to chemotherapeutic drugs used in HNSCC therapy, if any (data not shown).

All the abovementioned *EVI1*-associated phenotypes were investigated in at least four of the five cell line models (with technical aspects precluding analysis of the fifth model in case of the scratch assay and the endothelial cell displacement assay) and were observed in the majority of them. The absence of significant effects in some models and assays may partly be related to the different expression levels of exogenous EVI1 (Figures S1C,D and S7) and partly to intrinsic differences between the cell lines. Importantly, the observation of complementary phenotypes with the knock-down model confirmed the validity of the data obtained with the overexpression models. Curiously, however, in the endothelial cell displacement assay, only shEVI1-2, but not shEVI1-1, had an effect. Since both shRNAs caused substantial downregulation of EVI1 and behaved comparably in other assays, it appears unlikely that this discrepancy could be attributable to residual EVI1 activity or to an off-target effect of one of the shRNAs. In principle, it could be due to the fact that shEVI1-1, but not shEVI1-2, also targets a poorly characterized EVI1 splice variant termed EVI1Δ324, which is usually expressed along with full-length EVI1 [35] and was also present in the HNSCC cell lines investigated (Figure S1A). This would imply an antagonistic relationship between full-length EVI1 and EVI1Δ324 specifically with respect to the endothelial cell displacement phenotype, an assumption that would require further investigation. Notwithstanding, all the phenotypes described above were observed in at least three, and in up to five, of the five different cell line models, strongly supporting the relevance of *EVI1* to HNSCC biology. Our in vitro data therefore offer convincing explanations for the observations made with primary patient samples [31].

To gain insights into the molecular mechanisms mediating the effects of EVI1 in HNSCC, RNA-seq experiments were performed. As expected for a transcription factor, EVI1 regulated the expression of numerous genes in both CAL-33 and SCC-25 cells. Interestingly, the extent of the transcriptional alterations correlated with that of the functional effects, in that EVI1 caused the differential expression of 4.5 times more genes in the biologically more responsive CAL-33 cells compared with the SCC-25 cells. Nevertheless, 252 genes were regulated in the same direction in both cell lines. Among these, genes with roles in the observed biological phenotypes were enriched, supporting the expected importance of transcriptional regulation for the functional effects of EVI1.

Even though transcription factors are not considered straightforward drug targets, several therapeutic suggestions for *EVI1*-overexpressing malignancies have been made. Zhang et al. described a pyrrole−imidazole polyamide that specifically bound to EVI1 recognition motifs in DNA, and inhibited DNA binding and transcriptional regulation by EVI1 [36]. It also moderately inhibited the proliferation of and colony formation by hematopoietic cells expressing EVI1 or a leukemogenic EVI1 fusion protein [36]. Whether this substance is potent and specific enough for in vivo application remains to be determined. In AML, a prevalent mechanism for the aberrant expression of *EVI1* is its juxtaposition, via chromosomal rearrangements, to transcriptional super-enhancers [37,38]. Accordingly, the BET-bromodomain inhibitor JQ1 decreased *EVI1* mRNA levels, inhibited proliferation, and augmented the differentiation and apoptosis of AML cells overexpressing *EVI1* due to specific chromosome rearrangements [37]. While gene amplification was proposed as a potential mechanism for *EVI1* overexpression in ovarian cancer [12,27], activation of *EVI1*-associated distal enhancers was correlated with increased *EVI1* expression in pancreatic ductal adenocarcinoma, and JQ1 reduced *EVI1* expression in the corresponding organoids [11]. *MECOM* was amplified in 19% of HNSCC patients in the TCGA Firehose cohort [39], yet the mechanism(s) of upregulation of *EVI1* in HNSCC remain(s) to be determined. Should activation by super-enhancers also play a role in this entity, BET inhibitors may represent a therapeutic option. Thirdly, overexpression of *EVI1* was reported to augment sensitivity towards arsenic trioxide in in vitro models of myeloid malignancies and in patients with myelodysplastic syndrome [40]. Recently, arsenite loaded nanoparticles were shown to exhibit anti-tumor activity against *EVI1*-positive nasopharyngeal carcinoma cell lines both in vitro and in a xenograft model [9], raising the possibility that arsenic compounds may also be effective in solid tumors with high *EVI1* expression, including in HNSCC. Arsenic compounds and BET inhibitors are approved and under development, respectively, as anti-cancer therapeutics [41,42].

In summary, the data presented in this publication establish *EVI1* as novel relevant oncogene in HNSCC. Whether any of the approaches described above will be useful in the treatment of HNSCC with high *EVI1* expression remains to be determined.

## 4. Materials and Methods

### 4.1. Cell Culture

The HPV-negative human HNSCC cell lines CAL-33, CAL-27, SCC-25, SCC-4, BHY, FADU, and HN were acquired from the German Collection of Microorganisms and Cell Cultures GmbH, Braunschweig, Germany. CAL-33, CAL-27, BHY, and HN cells were cultured in DMEM (Thermo Fisher Scientific, Waltham, MA, USA) containing 10% FBS (Thermo Fisher Scientific), 1× penicillin–streptomycin (Sigma-Aldrich, St. Louis, MO, USA), and 2 mM L-glutamine (Thermo Fisher Scientific). For CAL-33 derivative cell lines obtained through transduction with inducible shRNA vectors, 1 µg/mL doxycycline (MP Biomedicals, Santa Ana, CA, USA) was added to the growth medium. SCC-25 cells were cultivated in DMEM/F12 (Thermo Fisher Scientific) supplemented with 20% FBS, 1× penicillin–streptomycin, 2 mM L-glutamine, and 1 mM sodium pyruvate (Thermo Fisher Scientific). SCC-4 cells were grown in DMEM/F12 containing 10% FBS, 1× penicillin–streptomycin, 2 mM L-glutamine, and 40 ng/mL hydrocortisone (Sigma-Aldrich). FADU cells were cultured in MEM (Thermo Fisher Scientific) containing 10% FBS and 1× penicillin–streptomycin. For the scratch assays, FBS concentrations were reduced to 0.2% (CAL-33 derivative lines), 1% (CAL-27 derivative lines), or 10% (SCC-25 derivative lines), which minimized cell proliferation while maintaining viability.

Phoenix GP cells (kindly provided by H. Stockinger, Medical University of Vienna, Austria) were grown in DMEM supplemented with 10% FBS and 1× penicillin–streptomycin-glutamine.

Isolation of HUVECs from consenting donors was approved by the ethics committee of the Medical University of Vienna (EK1621/2020), and was performed as described previously [43]. Briefly, umbilical veins were rinsed twice with HBSS (Lonza, Basel, Switzerland), filled with dispase (Corning, Bedford, MA, USA), and incubated for 10 min at 37 °C in a water bath. The detached endothelial cells were flushed out with HBSS, collected by centrifugation, and seeded in IMDM (Lonza) supplemented with 20% FBS, 1% Low Serum Growth Supplement (Thermo Fisher Scientific), 2 mM L-glutamine, and 1× penicillin–streptomycin. Culture dishes were coated with 1% gelatin. Only cells from passages 2 to 8 were used for the experiments.

All cells were cultured in a humidified incubator at 37 °C and 5% CO_2_. To passage cells, they were washed with phosphate-buffered saline (PBS; Thermo Fisher Scientific) and incubated with Trypsin-EDTA (0.05%; Thermo Fisher Scientific) at 37 °C until they detached from the dishes. Cell lines and HUVECs were tested regularly for *Mycoplasma* contamination using the MycoAlert™ Mycoplasma Detection Kit (Lonza).

### 4.2. Overexpression and Knock-Down of EVI1 in Human HNSCC Cell Lines

A retroviral vector expressing a codon-optimized version of the human *EVI1* cDNA (pMSCV-EVI1-eGFP) and lentiviral vectors containing doxycycline-inducible shRNAs targeting human *EVI1* (LT3REVIR-shEVI1-1, LT3REVIR-shEVI1-2) or the *renilla luciferase* gene as a control (LT3REVIR-shCtrl) have been described previously [19,44,45]. To generate viral particles containing pMSCV-EVI1-eGFP or the empty vector as a control, Phoenix GP cells were transfected with these vectors, along with the helper plasmids pMD2.G and pGagPol. In case of the shRNA vectors, the helper plasmids pMD2.G and psPAX2 were co-transfected. Transfections were performed using a standard calcium phosphate protocol. Supernatants containing viral particles were collected from transfected Phoenix GP cells, and any detached cells were removed by filtration through a 0.45 µM filter (VWR, Radnor, PA, USA). HNSCC cell lines were grown to 50% confluence in 6-well plates and transduced by centrifugation for 45 min at 250 g and 32 °C in the presence of retro- or lentiviral particles and 4 µg/mL polybrene (Sigma-Aldrich). Infection cycles were repeated after 24 and 48 h (retroviral transduction), or after 24 h (lentiviral transduction). Three days after the last transduction cycle, cells were sorted for fluorescence marker positivity (eGFP for pMSCV-based vectors, Venus for shRNA constructs) on a BD FACSAria™ Fusion cell sorter (BD Biosciences, Franklin Lakes, NJ, USA). Overexpression and knock-down of EVI1 were confirmed by immunoblot analysis.

### 4.3. Quantitative Real-Time Reverse Transcriptase PCR

Total RNA was isolated using TRIzol^®^ reagent (Thermo Fisher Scientific), and cDNA was synthesized using the LunaScript™ RT SuperMix Kit (NEB, Ipswich, MA, USA). Quantitative real-time reverse transcriptase PCR (qRT-PCR) was performed in triplicate on a StepOnePlus™ Real-Time PCR instrument using gene-specific TaqMan probes (*EVI1*: Hs00602795_m1; *β-2-microglobulin*: Hs99999907_m1), the TaqMan gene expression Mastermix (all from Thermo Fisher Scientific), and the instrument’s standard cycling protocol. *EVI1* expression levels were normalized to those of *β-2-microglobulin* and to a reference sample using the ΔΔC_T_ method [46].

### 4.4. Immunoblot Analysis

Protein extracts were prepared using a RIPA buffer (50 mM Tris/HCl (pH 8.0), 0.1% SDS, 0.5% sodium desoxycholate (all from Sigma-Aldrich), 150 mM NaCl (Carl Roth, Karlsruhe, Germany), and 1% Triton X-100 (Roche, Penzberg, Germany)) with 5% protease inhibitor (Sigma-Aldrich). Protein concentrations were determined using the Bradford assay (Bio-Rad Laboratories, Hercules, CA, USA). Samples were diluted to equal concentrations with 1× Roti^®^-Load 1 (Carl Roth). SDS-PAGE was performed following standard protocols. The Pierce™ Power Blotter (Thermo Fisher Scientific) was used for semi-dry protein transfer to PVDF membranes (Pall, Port Washington, NY, USA). Membranes were blocked with 5% non-fat dried milk (AppliChem, Darmstadt, Germany)/TBS-T (40 mM Tris/HCl (pH 7.6), 273 mM NaCl, 0.1% Tween^®^ 20 (Sigma-Aldrich)) for 1 h at room temperature. Incubation with primary antibodies was performed in blocking solution overnight at 4 °C, and incubation with secondary antibodies was performed in TBS-T for 1 h at room temperature. Immunoblots were developed with SuperSignal West Pico or Femto Chemiluminescent Substrates (Thermo Fisher Scientific), and signals were detected on a ChemiDoc Touch Imaging System (Bio-Rad). Bands were quantified using Image Lab v6 (Bio-Rad). The antibodies and the concentrations used are listed in Supplementary Table S2.

### 4.5. Cell Proliferation Assays

Real-time cell proliferation assays were performed using the xCELLigence system (OMNI Life Science, Bremen, Germany). For this, 2000 cells were seeded per well of an E-Plate 16 (Agilent Technologies, Santa Clara, CA, USA) in a complete growth medium. Assays were performed in quadruplicate. Impedance was measured every 15 min for 120 h, and the data were normalized to the 24 h time point.

To compare the proliferation of derivative HNSCC cell lines under normal and low-serum conditions, 2000 cells were seeded per well in a 96-well-plate (TPP, Trasadingen, Switzerland). Starting from the next day, resazurin (Sigma-Aldrich, 550 µM stock in RPMI 1640 (Thermo Fisher Scientific)) was added daily to a subset of wells to a final concentration of 55 µM. After incubation with resazurin for 6 h at 37 °C, fluorescence was measured (λ_ex_ = 507 nm, λ_em_ = 595 nm) on a Varioskan LUX microplate reader equipped with SkanIt v5 software (Thermo Fisher Scientific).

### 4.6. Colony Formation Assay

Two thousand cells from CAL-33, CAL-27, or SCC-25 derivative cell lines, or 500 cells from SCC-4 derivative cell lines were seeded per well of a 6-well plate and cultivated in their respective growth media for 11 days. For each sample, duplicates were prepared. Media were changed every 3 to 4 days. For staining, colonies were washed with PBS, fixed and permeabilized with 100% methanol (Fisher Scientific, Schwerte, Germany) for 5 min, washed again with PBS, and stained with 0.2% trypan blue (Merck, Darmstadt, Germany)/PBS for 5 min. Wells were washed with PBS, air-dried, and photographed. Colony numbers and sizes were quantified using ImageJ v1.53.

### 4.7. Scratch Assay

Cells were seeded into 6-well plates and cultivated in a complete growth medium for 1 to 3 days until they reached 90% confluence. Cell layers were washed twice with PBS, then media with reduced FBS concentrations were added (CAL-33: 0.2%; CAL-27: 1%; SCC-25: 10%), and cells were incubated for another 24 h. Scratches were made using a 200 µL pipet tip, and photographed after the indicated time periods. Scratch areas were quantified using Photoshop CS6 v13 (Adobe, San José, CA, USA), normalized to the 0 h time point, and converted to gap closure values by subtraction from 1. Therefore, gap closure values of 0 or 1 represent no or complete closure of the initial scratch area, respectively.

### 4.8. Transwell Migration and Invasion Assays

Cells were maintained in a serum-free medium for 24 h prior to the assays. Uncoated and Matrigel^®^-coated well inserts with PET membranes (8.0 µm pores, 24 wells; Corning, Corning, NY, USA) were used for the migration and invasion assays, respectively. To coat the membranes, 100 µL of 300 µg/mL Matrigel^®^ (Corning) in a serum-free growth medium was added per insert. After incubation for 3 h at 37 °C, the coating solution was removed. For both assay types, 250,000 cells in 200 µL of serum-free medium were seeded into the inserts (upper chamber) and 750 µL of complete growth medium was added into the wells (lower chamber). After 24 h, the inserts were removed from wells, and the cells were fixed with 4% formaldehyde (Sigma-Aldrich) /PBS for 2 min, permeabilized with methanol for 20 min, and stained with 0.2% trypan blue/PBS for 10 min. Cells on the upper side of the membrane were removed with a cotton swab. Photos of four representative regions of the membranes were taken, and cells were counted manually.

### 4.9. Spheroid Formation

Three thousand cells in 150 µL of complete growth medium were seeded per well of a 96-well Nunclon™ Sphera™ ultra-low attachment plate with round-bottomed wells (Thermo Fisher Scientific). Six technical replicates were performed. Plates were centrifuged for 15 min at 390 g and incubated in a cell culture incubator. Spheroids were photographed on the indicated days, and the cross-section area was determined using Photoshop CS6 v13.

### 4.10. Displacement of Endothelial Cells by Tumor Spheroids

HUVECs were prepared as described above (the “Cell Culture” section), seeded into gelatin-coated 4-well µ-slides (ibidi, Gräfelfing, Germany), and grown to confluence. They were stained with CellTracker™ Orange CMTMR Dye (Thermo Fisher Scientific; 1:2000 in complete HUVEC medium for experiments with *EVI1*-overexpressing cells) or CellTracker™ Deep Red Dye (Thermo Fisher Scientific, 1:1000 in serum-free IMDM for experiments with *EVI1* knock-down cells) for 3 or 1 h, respectively, at 37 °C. Tumor cell spheroids (8 to 16 per derivative cell line) were allowed to form for 2 (CAL-33 derivative lines) or 1 (CAL-27 and SCC-25 derivative lines) day(s) as described above (“Spheroid Formation” section). They were collected, pooled, washed twice with PBS, resuspended in 800 µL HUVEC medium without phenol red, added onto the confluent stained HUVEC monolayers, and dispersed by gently rocking the slides to ensure even distribution throughout the wells. The slides were placed into the incubation chamber of an IX83 live cell imaging microscope (Olympus, Tokyo, Japan), which maintained the cells at 37 °C in a humidified atmosphere with 5 % CO_2_. Spheroids were allowed to adhere to HUVECs for 1 h. Afterwards, circular discontinuities of the endothelial cell monolayer underneath the spheroids were imaged once per hour for 15 h in phase contrast and fluorescence channels with excitation at 561 nm and the Cy3 and Cy5 emission filters for CellTracker™ Orange and Deep Red, respectively. Gap areas for each time point were measured using ImageJ v1.53.

### 4.11. Statistics

At least three independent biological replicates were performed for each experiment (except for the endothelial cell displacement assay for CAL-27 derivative lines in Figure S6A, where *n* = 2). The number of technical replicates is indicated in the respective descriptions of each experiment type. Data are presented as means of the biological replicates ± SEM. Student’s two-sided *t*-test was used to assess the significance of differences between two independent groups. The one-sample *t*-test was used to compare an experimental sample to a control sample that had no variance because it was used for normalization. In cases where several comparisons with the same control sample were performed (i.e., experiments with knock-down cell lines), Bonferroni correction was applied to adjust for multiple testing. One-way ANOVA followed by Dunnett’s multiple comparison test was used for comparisons between multiple independent groups and a single control group, and two-way ANOVA followed by Bonferroni’s post-hoc test was used for multiple groups with two or more factors. These statistical tests were performed using GraphPad Prism 6 software (GraphPad Software, San Diego, CA, USA). Spearman’s correlation was calculated using the *cor.test* function in R v4.1.0. Linear mixed effects models were fitted for real-time cell proliferation experiments, and the significance of differences between the resulting curves was determined using the *anova.lme* function of the *nlme* (v3.1.153) package in R v4.1.0; *p*-values < 0.05 were considered statistically significant.

### 4.12. RNA-seq and Bioinformatic Analyses

RNA was extracted from 4 replicate cultures each of CAL-33_EVI1, CAL-33_vec, SCC-25_EVI1, and SCC-25_vec cells using TRIzol^®^, and the samples were submitted to the Genomics Core Facility of the Medical University of Vienna, Vienna, Austria, for further processing. RNA quality was checked on a Bioanalyzer 2100 (Agilent Technologies). RNA-seq library preparation was performed using the QuantSeq 3′ mRNA-Seq Library Prep Kit FWD (Lexogen, Greenland, NH, USA) according to the manufacturer’s instructions. In order to avoid distortion of the gene expression values due to over-cycling, the optimal number of PCR cycles for library preparation was determined by qPCR according to the library preparation manual. Library fragment size was checked on a Bioanalyzer 2100 using the High Sensitivity DNA Kit (Agilent Technologies), and dsDNA was quantified using the Qubit dsDNA HS Assay (Thermo Fisher Scientific); 75 bp single-end sequencing of pooled libraries was performed on a NextSeq500 instrument (Illumina, San Diego, CA, USA).

Fastq files containing unmapped reads were generated using Illumina bcl2fastq Conversion software (v2.19.1.403). Adapter trimming and read filtering were performed using cutadapt (v1.15). Reads were aligned to the human reference genome build GRCh38 with Gencode 29 annotations using STAR aligner (v2.6.1a) in 2-pass mode. The expression of codon-optimized *EVI1* was verified through alignment to a modified GRCh38 STAR index containing the codon-optimized *EVI1* sequence. Differentially expressed genes were identified with DESeq2 (v1.22.2) using an FDR of <0.1 as the cut-off for significance. Overlaps of genes up- or down-regulated consistently between cell lines were visualized as Venn diagrams. Heatmaps were generated using the ClustVis tool [47]. Gene Ontology enrichment analyses were performed using ShinyGO v0.61 [48]. RNA-seq data were deposited in the Gene Expression Omnibus (accession number GSE187454).

RNA-seq data from the TCGA HNSCC Firehose legacy cohort were obtained from the cBioPortal database [49,50].

## Data Availability

RNA-seq data were deposited in the Gene Expression Omnibus (accession number GSE187454).

## Supplementary Materials

The following supporting information can be downloaded at: https://www.mdpi.com/article/10.3390/ijms23031050/s1.

## References

[1] Alsahafi E., Begg K., Amelio I., Raulf N., Lucarelli P., Sauter T., Tavassoli M. (2019). Clinical update on head and neck cancer: Molecular biology and ongoing challenges. Cell Death Dis..

[2] Cramer J.D., Burtness B., Le Q.T., Ferris R.L. (2019). The changing therapeutic landscape of head and neck cancer. Nat. Rev. Clin. Oncol..

[3] Wieser R. (2007). The oncogene and developmental regulator EVI1: Expression, biochemical properties, and biological functions. Gene.

[4] Birdwell C., Fiskus W., Kadia T.M., DiNardo C.D., Mill C.P., Bhalla K.N. (2021). EVI1 dysregulation: Impact on biology and therapy of myeloid malignancies. Blood Cancer J..

[5] Morishita K., Parker D.S., Mucenski M.L., Jenkins N.A., Copeland N.G., Ihle J.N. (1988). Retroviral activation of a novel gene encoding a zinc finger protein in IL-3-dependent myeloid leukemia cell lines. Cell.

[6] Bard-Chapeau E.A., Jeyakani J., Kok C.H., Muller J., Chua B.Q., Gunaratne J., Batagov A., Jenjaroenpun P., Kuznetsov V.A., Wei C.L. (2012). Ecotopic viral integration site 1 (EVI1) regulates multiple cellular processes important for cancer and is a synergistic partner for FOS protein in invasive tumors. Proc. Natl. Acad. Sci. USA.

[7] Glass C., Wuertzer C., Cui X., Bi Y., Davuluri R., Xiao Y.Y., Wilson M., Owens K., Zhang Y., Perkins A. (2013). Global Identification of EVI1 Target Genes in Acute Myeloid Leukemia. PLoS ONE.

[8] Mateo F., Arenas E.J., Aguilar H., Serra-Musach J., de Garibay G.R., Boni J., Maicas M., Du S., Iorio F., Herranz-Ors C. (2017). Stem cell-like transcriptional reprogramming mediates metastatic resistance to mTOR inhibition. Oncogene.

[9] Lu Y., Liang Y., Zheng X., Deng X., Huang W., Zhang G. (2019). EVI1 promotes epithelial-to-mesenchymal transition, cancer stem cell features and chemo-/radioresistance in nasopharyngeal carcinoma. J. Exp. Clin. Cancer Res..

[10] Bleu M., Mermet-Meillon F., Apfel V., Barys L., Holzer L., Bachmann Salvy M., Lopes R., Barbosa I.A.M., Delmas C., Hinniger A. (2021). PAX8 and MECOM are interaction partners driving ovarian cancer. Nat. Commun..

[11] Kim H.R., Yim J., Yoo H.B., Lee S.E., Oh S., Jung S., Hwang C.I., Shin D.M., Kim T., Yoo K.H. (2021). EVI1 activates tumor-promoting transcriptional enhancers in pancreatic cancer. NAR Cancer.

[12] Wang Z., Li Y., Wang N., Li P., Kong B., Liu Z. (2021). EVI1 overexpression promotes ovarian cancer progression by regulating estrogen signaling. Mol. Cell Endocrinol..

[13] Hoyt P., Bartholomew C., Davis A., Yutzey K., Gamer L., Potter S., Ihle J., Mucenski M. (1997). The Evi1 proto-oncogene is required at midgestation for neural, heart, and paraxial mesenchyme development. Mech. Dev..

[14] Goyama S., Yamamoto G., Shimabe M., Sato T., Ichikawa M., Ogawa S., Chiba S., Kurokawa M. (2008). Evi-1 is a critical regulator for hematopoietic stem cells and transformed leukemic cells. Cell Stem Cell.

[15] Kataoka K., Sato T., Yoshimi A., Goyama S., Tsuruta T., Kobayashi H., Shimabe M., Arai S., Nakagawa M., Imai Y. (2011). Evi1 is essential for hematopoietic stem cell self-renewal, and its expression marks hematopoietic cells with long-term multilineage repopulating activity. J. Exp. Med..

[16] Haas K., Kundi M., Sperr W.R., Esterbauer H., Ludwig W.D., Ratei R., Koller E., Gruener H., Sauerland C., Fonatsch C. (2008). Expression and prognostic significance of different mRNA 5′-end variants of the oncogene EVI1 in 266 patients with de novo AML: EVI1 and MDS1/EVI1 overexpression both predict short remission duration. Genes Chromosomes Cancer.

[17] Groschel S., Lugthart S., Schlenk R.F., Valk P.J., Eiwen K., Goudswaard C., van Putten W.J., Kayser S., Verdonck L.F., Lubbert M. (2010). High EVI1 expression predicts outcome in younger adult patients with acute myeloid leukemia and is associated with distinct cytogenetic abnormalities. J. Clin. Oncol..

[18] Groschel S., Schlenk R.F., Engelmann J., Rockova V., Teleanu V., Kuhn M.W., Eiwen K., Erpelinck C., Havermans M., Lubbert M. (2013). Deregulated expression of EVI1 defines a poor prognostic subset of MLL-rearranged acute myeloid leukemias: A study of the German-Austrian Acute Myeloid Leukemia Study Group and the Dutch-Belgian-Swiss HOVON/SAKK Cooperative Group. J. Clin. Oncol..

[19] Nguyen C.H., Bauer K., Hackl H., Schlerka A., Koller E., Hladik A., Stoiber D., Zuber J., Staber P.B., Hoelbl-Kovacic A. (2019). All-trans retinoic acid enhances, and a pan-RAR antagonist counteracts, the stem cell promoting activity of EVI1 in acute myeloid leukemia. Cell Death Dis..

[20] Hou A., Zhao L., Zhao F., Wang W., Niu J., Li B., Zhou Z., Zhu D. (2016). Expression of MECOM is associated with unfavorable prognosis in glioblastoma multiforme. OncoTargets Ther..

[21] He D., Wu L., Li X., Liu X., Ma P., Juang Y. (2019). Ecotropic virus integration-1 and calreticulin as novel prognostic markers in triple-negative breast cancer: A retrospective cohort study. Oncol. Lett..

[22] Palomero L., Bodnar L., Mateo F., Herranz-Ors C., Espin R., Garcia-Varelo M., Jesiotr M., Ruiz de Garibay G., Casanovas O., Lopez J.I. (2020). EVI1 as a Prognostic and Predictive Biomarker of Clear Cell Renal Cell Carcinoma. Cancers.

[23] Xu X., Liu S., Ji X. (2017). Overexpression of ecotropic viral integration site-1 is a prognostic factor of lung squamous cell cancer. Onco Targets Ther..

[24] Tanaka M., Shibahara J., Ishikawa S., Ushiku T., Morikawa T., Shinozaki-Ushiku A., Hayashi A., Misumi K., Tanaka A., Katoh H. (2019). EVI1 expression is associated with aggressive behavior in intrahepatic cholangiocarcinoma. Virchows Arch..

[25] Zhang X.M., Liu Z.L., Qiu B., Xu Y.F., Pan C., Zhang Z.L. (2020). Downregulation of EVI1 Expression Inhibits Cell Proliferation and Induces Apoptosis in Hilar Cholangiocarcinoma via the PTEN/AKT Signalling Pathway. J. Cancer.

[26] Queisser A., Hagedorn S., Wang H., Schaefer T., Konantz M., Alavi S., Deng M., Vogel W., von Massenhausen A., Kristiansen G. (2017). Ecotropic viral integration site 1, a novel oncogene in prostate cancer. Oncogene.

[27] Nanjundan M., Nakayama Y., Cheng K.W., Lahad J., Liu J., Lu K., Kuo W.L., Smith-McCune K., Fishman D., Gray J.W. (2007). Amplification of MDS1/EVI1 and EVI1, located in the 3q26.2 amplicon, is associated with favorable patient prognosis in ovarian cancer. Cancer Res..

[28] Tanaka M., Suzuki H.I., Shibahara J., Kunita A., Isagawa T., Yoshimi A., Kurokawa M., Miyazono K., Aburatani H., Ishikawa S. (2014). EVI1 oncogene promotes KRAS pathway through suppression of microRNA-96 in pancreatic carcinogenesis. Oncogene.

[29] Liu Y., Chen L., Ko T.C., Fields A.P., Thompson E.A. (2006). Evi1 is a survival factor which conveys resistance to both TGFbeta- and taxol-mediated cell death via PI3K/AKT. Oncogene.

[30] Wu L., Wang T., He D., Li X., Jiang Y. (2019). EVI1 acts as an oncogene and positively regulates calreticulin in breast cancer. Mol. Med. Rep..

[31] Idel C., Ribbat-Idel J., Kuppler P., Krupar R., Offermann A., Vogel W., Rades D., Kirfel J., Wollenberg B., Perner S. (2020). EVI1 as a Marker for Lymph Node Metastasis in HNSCC. Int. J. Mol. Sci..

[32] Ishiguro T., Ohata H., Sato A., Yamawaki K., Enomoto T., Okamoto K. (2017). Tumor-derived spheroids: Relevance to cancer stem cells and clinical applications. Cancer Sci..

[33] Buonamici S., Li D., Chi Y., Zhao R., Wang X., Brace L., Ni H., Saunthararajah Y., Nucifora G. (2004). EVI1 induces myelodysplastic syndrome in mice. J. Clin. Investig..

[34] Bindels E.M., Havermans M., Lugthart S., Erpelinck C., Wocjtowicz E., Krivtsov A.V., Rombouts E., Armstrong S.A., Taskesen E., Haanstra J.R. (2012). EVI1 is critical for the pathogenesis of a subset of MLL-AF9-rearranged AMLs. Blood.

[35] Kilbey A., Bartholomew C. (1998). Evi-1 ZF1 DNA binding activity and a second distinct transcriptional repressor region are both required for optimal transformation of Rat1 fibroblasts. Oncogene.

[36] Zhang Y., Sicot G., Cui X., Vogel M., Wuertzer C.A., Lezon-Geyda K., Wheeler J., Harki D.A., Muzikar K.A., Stolper D.A. (2011). Targeting a DNA binding motif of the EVI1 protein by a pyrrole-imidazole polyamide. Biochemistry.

[37] Groschel S., Sanders M.A., Hoogenboezem R., de Wit E., Bouwman B.A.M., Erpelinck C., van der Velden V.H.J., Havermans M., Avellino R., van Lom K. (2014). A single oncogenic enhancer rearrangement causes concomitant EVI1 and GATA2 deregulation in leukemia. Cell.

[38] Ottema S., Mulet-Lazaro R., Erpelinck-Verschueren C., van Herk S., Havermans M., Arricibita Varea A., Vermeulen M., Beverloo H.B., Groschel S., Haferlach T. (2021). The leukemic oncogene EVI1 hijacks a MYC super-enhancer by CTCF-facilitated loops. Nat. Commun..

[39] cBioPortal for Cancer Genomics: Head and Neck Squamous Cell Carcinoma (TCGA, Firehose Legacy). https://www.cbioportal.org/study/summary?id=hnsc_tcga.

[40] Raza A., Buonamici S., Lisak L., Tahir S., Li D.L., Imran M., Chaudary N.I., Pervaiz H., Gallegos J.A., Alvi M.I. (2004). Arsenic trioxide and thalidomide combination produces multi-lineage hematological responses in myelodysplastic syndromes patients, particularly in those with high pre-therapy EVI1 expression. Leuk Res..

[41] Sanz M.A., Fenaux P., Tallman M.S., Estey E.H., Lowenberg B., Naoe T., Lengfelder E., Dohner H., Burnett A.K., Chen S.J. (2019). Management of acute promyelocytic leukemia: Updated recommendations from an expert panel of the European LeukemiaNet. Blood.

[42] Bechter O., Schoffski P. (2020). Make your best BET: The emerging role of BET inhibitor treatment in malignant tumors. Pharmacol. Ther..

[43] Schossleitner K., O’Mahony C., Brandstatter S., Haslinger M.J., Demuth S., Fechtig D., Petzelbauer P. (2019). Differences in biocompatibility of microneedles from cyclic olefin polymers with human endothelial and epithelial skin cells. J. Biomed. Mater. Res. A.

[44] Steinleitner K., Rampetsreiter P., Koffel R., Ramanathan G., Mannhalter C., Strobl H., Wieser R. (2012). EVI1 and MDS1/EVI1 expression during primary human hematopoietic progenitor cell differentiation into various myeloid lineages. Anticancer Res..

[45] Fellmann C., Hoffmann T., Sridhar V., Hopfgartner B., Muhar M., Roth M., Lai D.Y., Barbosa I.A., Kwon J.S., Guan Y. (2013). An optimized microRNA backbone for effective single-copy RNAi. Cell Rep..

[46] Livak K.J., Schmittgen T.D. (2001). Analysis of relative gene expression data using real-time quantitative PCR and the 2(T)(-Delta Delta C) method. Methods.

[47] Metsalu T., Vilo J. (2015). ClustVis: A web tool for visualizing clustering of multivariate data using Principal Component Analysis and heatmap. Nucleic Acids Res..

[48] Ge S.X., Jung D., Yao R. (2020). ShinyGO: A graphical gene-set enrichment tool for animals and plants. Bioinformatics.

[49] Cerami E., Gao J., Dogrusoz U., Gross B.E., Sumer S.O., Aksoy B.A., Jacobsen A., Byrne C.J., Heuer M.L., Larsson E. (2012). The cBio cancer genomics portal: An open platform for exploring multidimensional cancer genomics data. Cancer Discov..

[50] Gao J., Aksoy B.A., Dogrusoz U., Dresdner G., Gross B., Sumer S.O., Sun Y., Jacobsen A., Sinha R., Larsson E. (2013). Integrative analysis of complex cancer genomics and clinical profiles using the cBioPortal. Sci. Signal..

