# Supplementary material for: EVI1 Promotes the Proliferation and Invasive Properties of Human Head and Neck Squamous Cell Carcinoma Cells
Source: Int J Mol Sci. 2022 Jan 19;23(3):1050. doi: 10.3390/ijms23031050 (PMC8835242; doi:10.3390/ijms23031050)
Supplement: Supplementary file 1 [file ijms-23-01050-s001.zip › ijms-1537380-Supplementary.pdf]

Supplementary information for the manuscript:

# *EVI1* promotes proliferation and invasive properties of human head and neck squamous cell carcinoma cells

by A. M. Grandits *et al.*

**This supplementary information includes:**

1. Supplementary Table S1 (caption only)
2. Supplementary Table S2
3. Supplementary Figure Legends
4. Supplementary Figures S1 to S7

**Supplementary Table S1:** EVI1 regulates the expression of numerous genes in HNSCC cells. Quadruplicate cultures of CAL-33_EVI1, CAL-33_vec, SCC-25_EVI1, and SCC-25_vec were subjected to RNA sequencing. (A) List of 3,377 genes differentially expressed at a false discovery rate (FDR) < 0.1 between CAL-33_EVI1 and CAL-33_vec cells. (B) List of 751 genes differentially expressed at an FDR < 0.1 between SCC-25_EVI1 and SCC-25_vec cells. (C) List of 252 genes whose expression was significantly altered in the same direction upon experimental expression of EVI1 in CAL-33 and SCC-25 cells (100 genes that were regulated in opposite directions in the two cell lines are not listed). (D) Gene Ontology enrichment analysis of 252 genes commonly affected by EVI1 in CAL-33 and SCC-25 cells. Analyses were performed using ShinyGO v0.61. (E) List of 39 genes regulated concordantly by EVI1 in CAL-33 and SCC-25 cells, and correlated with *EVI1* expression in the corresponding direction in 522 HNSCC patients from the TCGA Firehose cohort.

Table is provided in a separate file.

**Supplementary Table S2:** List of antibodies used for immunoblot analysis.

| Target | Company | Conjugate | Clone/ cat.no. | Dilution |
|---|---|---|---|---|
| Human EVI1 | Cell Signaling Technology | - | C50E12 | 1:1.000 |
| Human GAPDH | Cell Signaling Technology | - | 14C10 | 1:30.000 |
| Rabbit IgG | Jackson ImmunoResearch | Horseradish peroxidase | 111035008 | 1:10.000 |

## SUPPLEMENTARY FIGURE LEGENDS

**Supplementary Figure S1: Expression of *EVI1* in native and transduced HNSCC cell lines.** (A) EVI1 protein levels in seven HNSCC cell lines were determined by immunoblot analysis; GAPDH was used as a loading control. Full size blot corresponding to Figure 1A. (B) EVI1 mRNA levels in seven human HNSCC cell lines were determined by qRT-PCR, and normalized to *β-2-microglobulin* mRNA levels and to CAL-27 cells using the $\Delta\Delta C_T$ method. Means + SEM, n = 3. (C-E) CAL-27 (C), SCC-25 (D), and SCC-4 (E) cells were transduced with a retroviral vector containing the human *EVI1* cDNA (EVI1) or with empty vector as a control (vec). Overexpression of EVI1 was confirmed by immunoblot analysis. Left panels, representative immunoblots; right panels, quantifications. Means + SEM, n = 3. * $p < 0.05$, n.s., not significant, one sample *t*-test.

**Supplementary Figure S2: EVI1 promotes proliferation of CAL-27, SCC-25, and SCC-4 cells.** (A-C) Proliferation of CAL-27_EVI1 and CAL-27_vec cells (A), SCC-25_EVI1 and SCC-25_vec cells (B), or SCC-4_EVI1 and SCC-4_vec cells (C) was monitored in real time for 120 h. Impedance values determined by the xCELLigence system were normalized to the 24 h time point. Means ± SEM, n = 3. *** $p < 0.001$, *anova.lme* function of the R package *nlme.* (D-F) Colony formation. CAL-27_EVI1 and CAL-27_vec cells (D), SCC-25_EVI1 and SCC-25_vec cells (E), or SCC-4_EVI1 and SCC-4_vec cells (F) were seeded at low densities into 6-well-plates and stained with trypan blue after 11 days. Top panels, quantifications of colony sizes and colony numbers; bottom panels, representative well areas. Means + SEM, n = 3. * $p < 0.05$, Student's two-sided *t*-test.

**Supplementary Figure S3: EVI1 increases the size of CAL-27 spheroids.** Tumor spheroids from CAL-27_EVI1 and CAL-27_vec cells (A), SCC-25_EVI1 and SCC-25_vec cells (B), or SCC-4_EVI1 and SCC-4_vec cells (C) were allowed to form over a period of 3 days. Top panels, quantifications of spheroid sizes; bottom panels, representative spheroids. Means + SEM, n = 3. * p < 0.05, Student's two-sided *t*-test.

**Supplementary Figure S4: EVI1 enhances migration of CAL-33, CAL-27, and SCC-25 cells (scratch assays).** (A,B) Scratch assay of CAL-33 derivative cell lines in regular growth medium. CAL-33_EVI1 and CAL-33_vec cells (A), or CAL-33_shEVI1-1, CAL-33_shEVI1-2, and CAL-33_shCtrl cells (B) were grown to confluence. Scratches were introduced and gap closure was monitored at the indicated time points. Top panels, quantifications; bottom panels, representative experiments. Means + SEM, n = 3. * p < 0.05, ** p < 0.01, *** p < 0.001, two-way ANOVA followed by Bonferroni's post-hoc test. (C-E) Impact of reduced fetal bovine serum (FBS) concentrations on cell proliferation. CAL-33_EVI1 and CAL-33_vec cells (C), CAL-27_EVI1 and CAL-27_vec cells (D), or SCC-25_EVI1 and SCC-25_vec cells (E) were cultivated under regular or reduced FBS conditions as indicated. Metabolic activity as a proxy for cell numbers was determined on days 0, 1, 2, and 3 using the Alamar Blue assay. Means ± SEM, n = 3. (F,G) Scratch assays under reduced serum conditions. CAL-27_EVI1 and CAL-27_vec cells (F) or SCC-25_EVI1 and SCC-25_vec cells (G) were grown to confluence in regular growth medium. The medium was changed to 1% FBS (F) or 10% FBS (G), scratches were introduced 24 h later, and gap closure was monitored at the indicated time points. Top panels, quantifications; bottom panels, representative experiments. Means + SEM, n = 3. * p < 0.05, *** p < 0.001, two-way ANOVA followed by Bonferroni's post-hoc test. SCC-4 derivative cell lines strongly adhered to the culture plate surface, precluding introduction of an evaluable scratch.

**Supplementary Figure S5: EVI1 enhances migration and invasion of HNSCC cell lines (Transwell assays).** (A-C) Transwell migration assay. Serum-starved CAL-27_EVI1 and CAL-27_vec cells (A), SCC-25_EVI1 and SCC-25_vec cells (B), or SCC-4_EVI1 and SCC-4_vec cells (C) were seeded into uncoated Transwell inserts and allowed to migrate towards medium containing 10% (A,C) or 20% (B) fetal bovine serum (FBS). After 24 h, cells at the bottom of the Transwell membranes were stained with trypan blue. (D-H) Transwell invasion assay. Serum-starved CAL-33_EVI1 and CAL-33_vec cells (D), CAL-33_shEVI1-1, CAL-33_shEVI1-2, and CAL-33_shCtrl cells (E), CAL-27_EVI1 and CAL-27_vec cells (F), SCC-25_EVI1 and SCC-25_vec cells (G), or SCC-4_EVI1 and SCC-4_vec cells (H) were seeded into Matrigel®-coated Transwell inserts. Cells were allowed to invade Matrigel to migrate towards medium containing 10% (D-F,H) or 20% (G) FBS. After 24 h, cells at the bottom of the Transwell membranes were stained with trypan blue. (A-H) Left panels: quantifications; right panels, images of representative experiments. Means + SEM, n = 3. * $p < 0.05$, one sample *t*-test (A-D,F-G), one sample *t*-test with Bonferroni correction for multiple testing (E).

**Supplementary Figure S6: EVI1 enhances displacement of endothelial cells by SCC-25 derived spheroids.** (A,B) Spheroids were generated from CAL-27_EVI1 and CAL-27_vec cells (A) or SCC-25_EVI1 and SCC-25_vec cells (B). They were placed onto confluent HUVECs that had been stained with CellTracker™ Orange CMTMR Dye, and HUVEC displacement was followed by live-cell imaging. Top panels: quantifications; bottom panels, images of representative experiments. Means + SEM, n = 2 (A) or 3 (B). Differences of gap areas were tested for statistical significance after 15 h. ** $p < 0.01$, Student's two-sided *t*-test.

**Supplementary Figure S7: Experimental expression of codon-optimized *EVI1* in CAL-33 and SCC-25 cells can be verified by RNA-seq, and leads to down-regulation of endogenous *EVI1* mRNA.** Left panel: Sequence counts of the experimentally expressed, codon-optimized *EVI1* mRNA, determined through alignment to a modified GRCh38 STAR index containing the corresponding sequence. Right panel: sequence counts of the endogenous *EVI1* mRNA. Please note that the net effect of these alterations is an up-regulation of EVI1 at the protein level (Figure 1B, S1D).

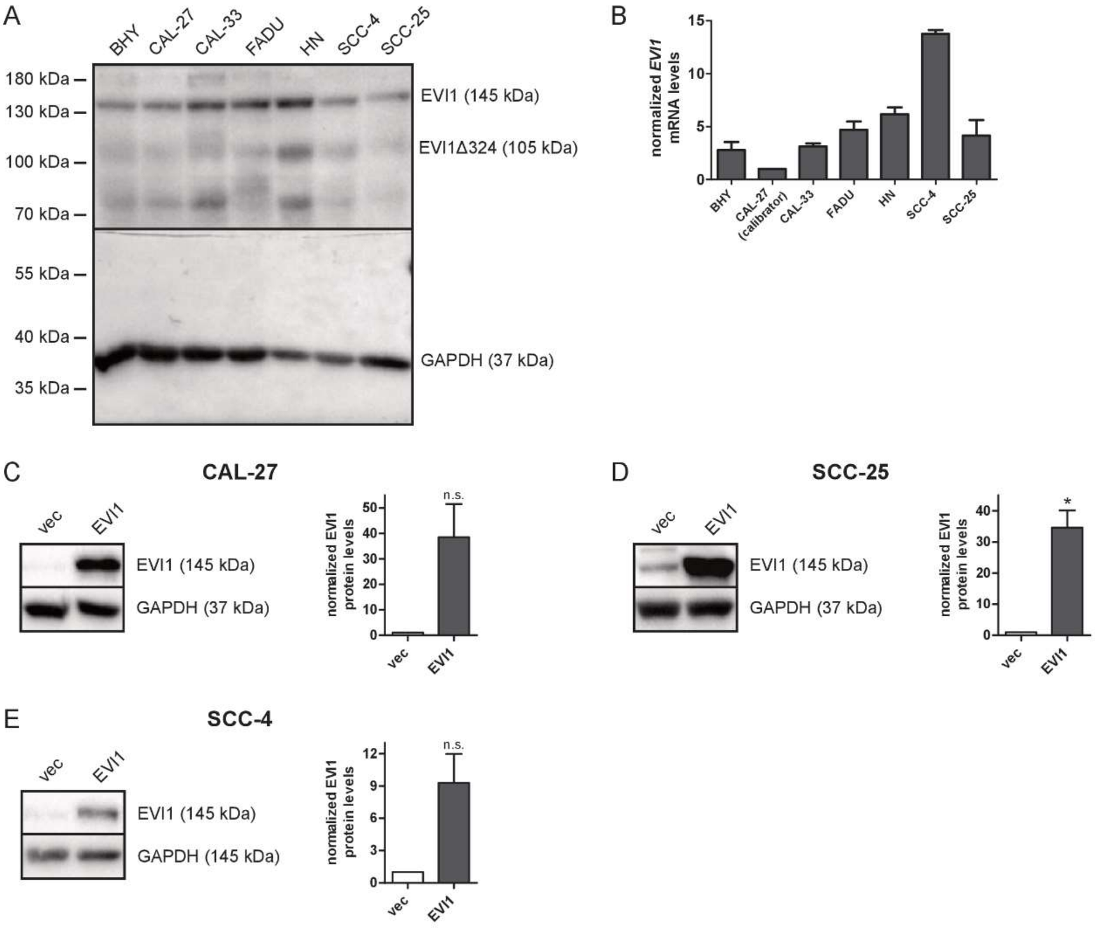
A
BHY
CAL-27
CAL-33
FADU
HN
SCC-4
SCC-25
180 kDa
130 kDa
100 kDa
70 kDa
55 kDa
40 kDa
35 kDa
EVI1 (145 kDa)
EVI1Δ324 (105 kDa)
GAPDH (37 kDa)
B
normalized EVI1 mRNA levels
CAL-27 (calibrator)
C
CAL-27
vec
EVI1
EVI1 (145 kDa)
GAPDH (37 kDa)
normalized EVI1 protein levels
n.s.
D
SCC-25
*
E
SCC-4
GAPDH (145 kDa)

**Figure S1**

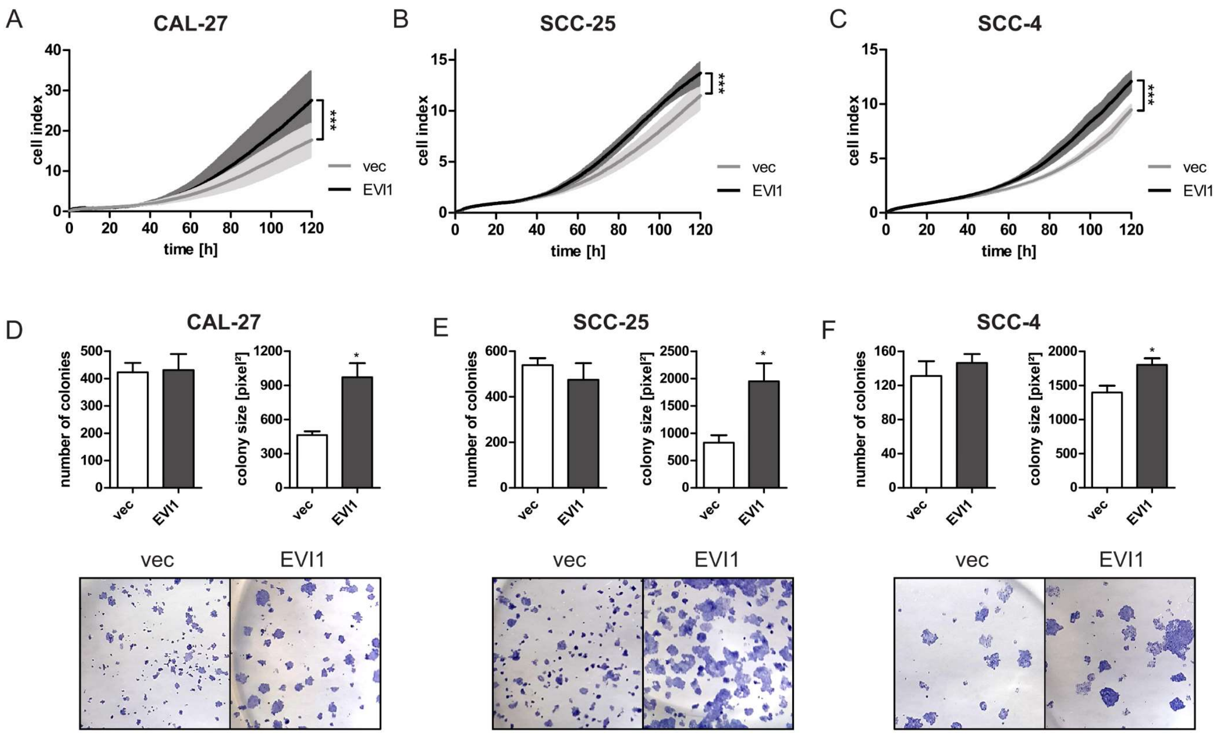
A
CAL-27
B
SCC-25
C
SCC-4
cell index
time [h]
vec
EVI1
***
D
E
F
number of colonies
colony size [pixel²]
vec
EVI1
*

**Figure S2**

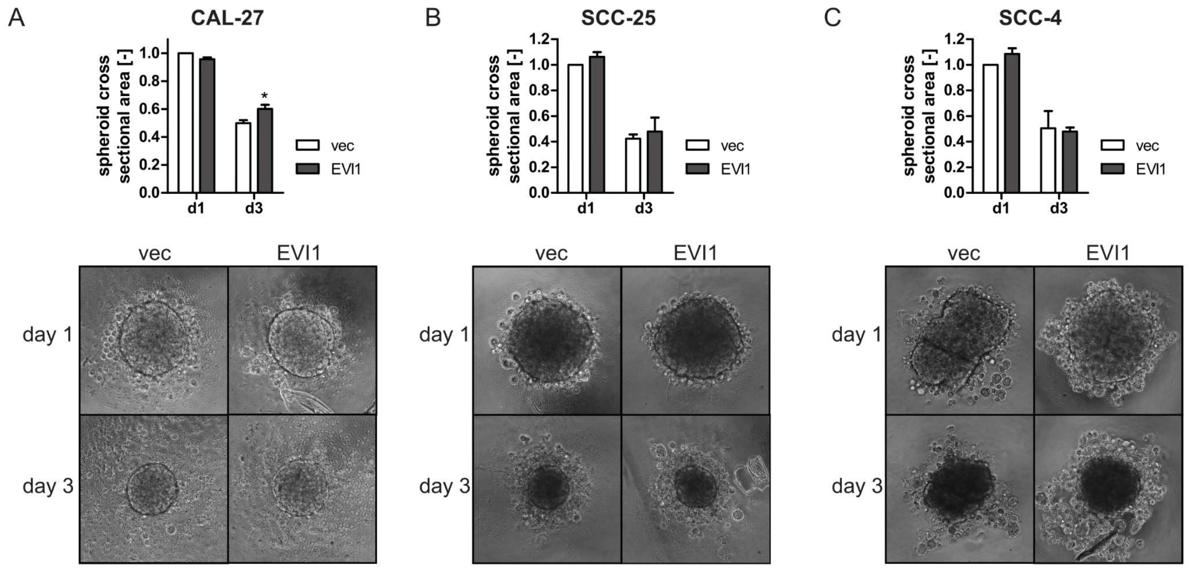
A
CAL-27
spheroid cross sectional area [-]
1.0
0.8
0.6
0.4
0.2
0.0
*
d1
d3
vec
EVI1
B
SCC-25
1.2
C
SCC-4
vec
EVI1
day 1
day 3

**Figure S3**

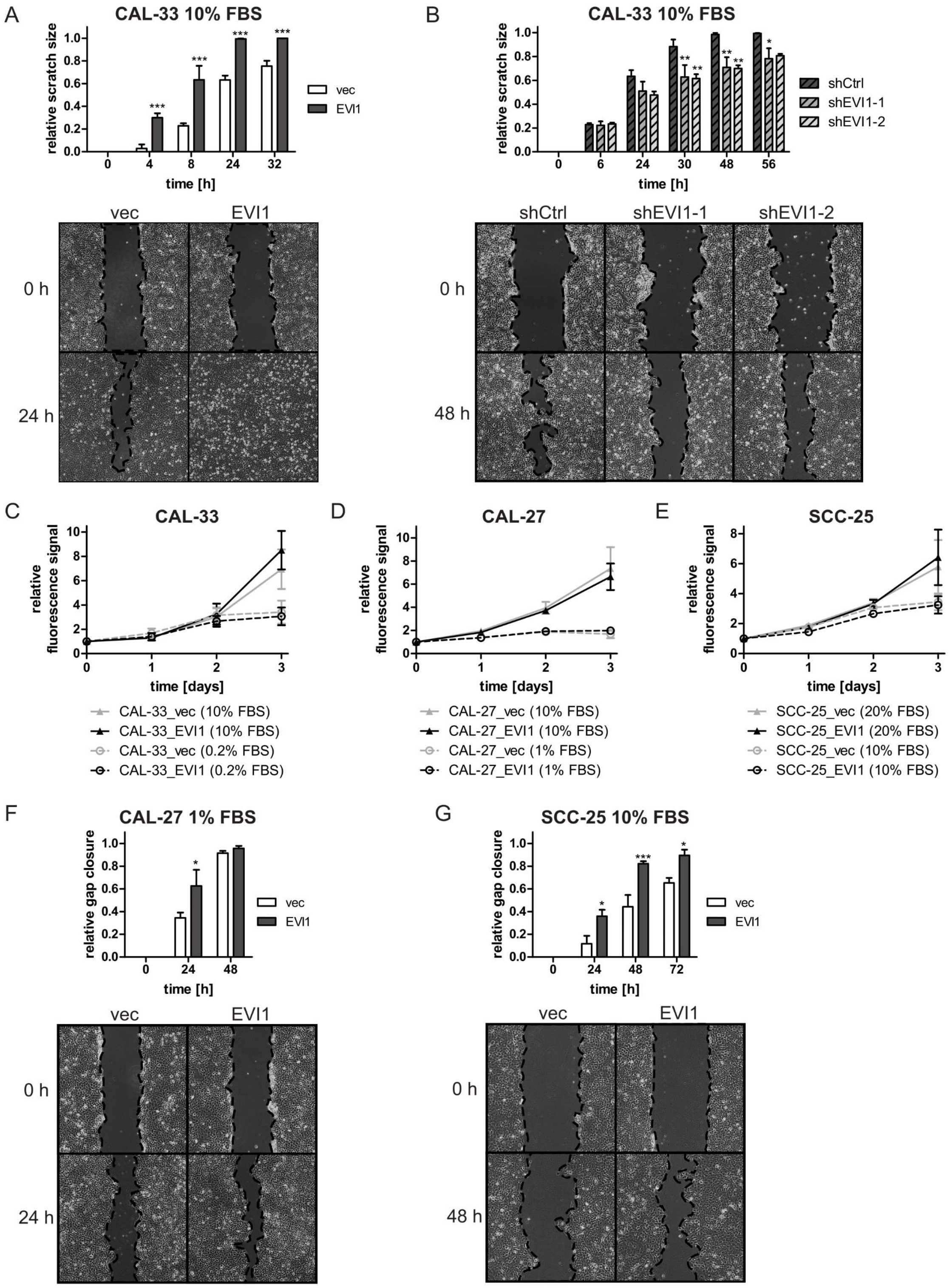
A
CAL-33 10% FBS
relative scratch size
time [h]
vec
EVI1
0 h
24 h
B
CAL-33 10% FBS
relative scratch size
time [h]
shCtrl
shEVI1-1
shEVI1-2
0 h
48 h
C
CAL-33
relative fluorescence signal
time [days]
CAL-33_vec (10% FBS)
CAL-33_EVI1 (10% FBS)
CAL-33_vec (0.2% FBS)
CAL-33_EVI1 (0.2% FBS)
D
CAL-27
CAL-27_vec (10% FBS)
CAL-27_EVI1 (10% FBS)
CAL-27_vec (1% FBS)
CAL-27_EVI1 (1% FBS)
E
SCC-25
SCC-25_vec (20% FBS)
SCC-25_EVI1 (20% FBS)
SCC-25_vec (10% FBS)
SCC-25_EVI1 (10% FBS)
F
CAL-27 1% FBS
relative gap closure
time [h]
vec
EVI1
0 h
24 h
G
SCC-25 10% FBS
relative gap closure
time [h]
vec
EVI1
0 h
48 h

**Figure S4**

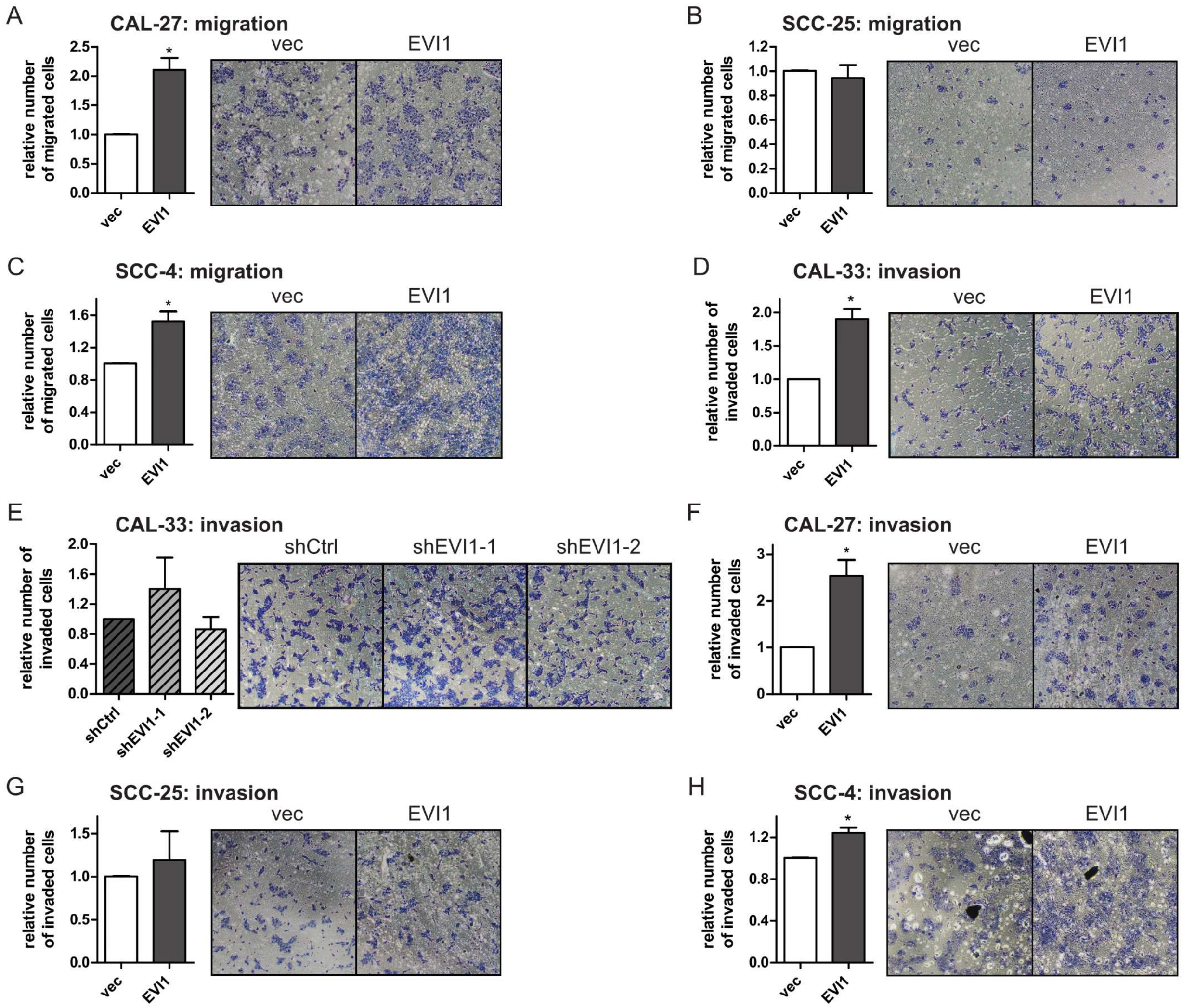
A
CAL-27: migration
relative number of migrated cells
vec
EVI1
B
SCC-25: migration
relative number of migrated cells
vec
EVI1
C
SCC-4: migration
relative number of migrated cells
vec
EVI1
D
CAL-33: invasion
relative number of invaded cells
vec
EVI1
E
CAL-33: invasion
relative number of invaded cells
shCtrl
shEVI1-1
shEVI1-2
F
CAL-27: invasion
relative number of invaded cells
vec
EVI1
G
SCC-25: invasion
relative number of invaded cells
vec
EVI1
H
SCC-4: invasion
relative number of invaded cells
vec
EVI1

**Figure S5**

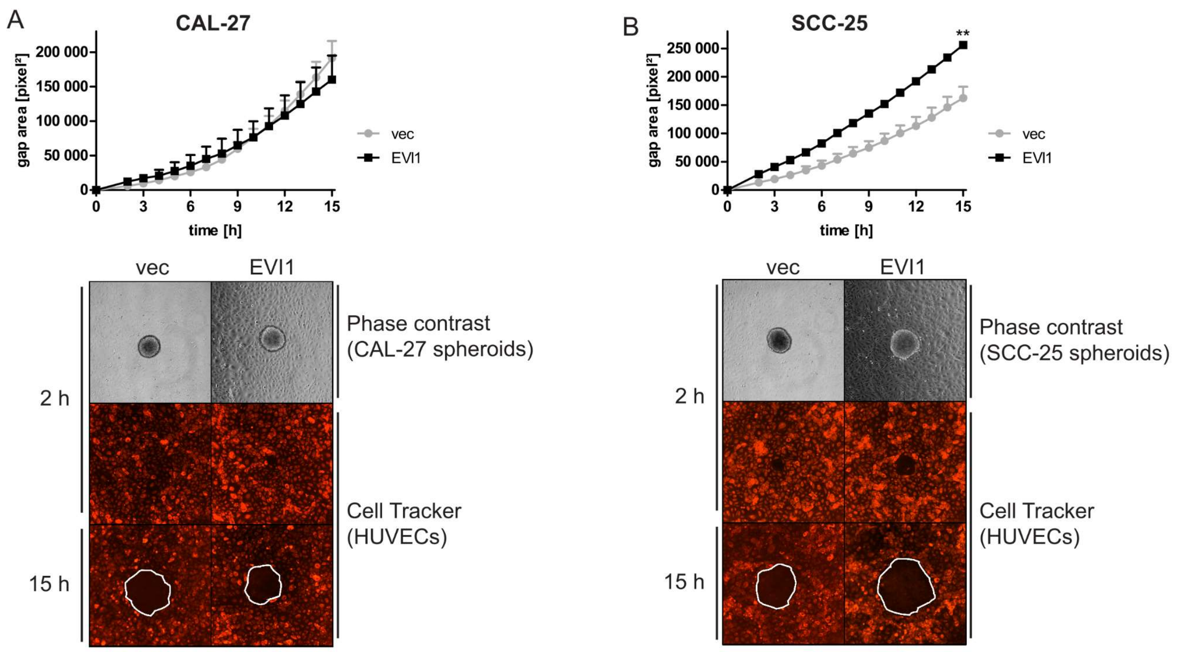
A
CAL-27
gap area [pixel²]
200 000
150 000
100 000
50 000
0
0
3
6
9
12
15
time [h]
vec
EVI1
B
SCC-25
**
250 000
200 000
150 000
100 000
50 000
vec
EVI1
2 h
15 h
Phase contrast
(CAL-27 spheroids)
(SCC-25 spheroids)
Cell Tracker
(HUVECs)

**Figure S6**

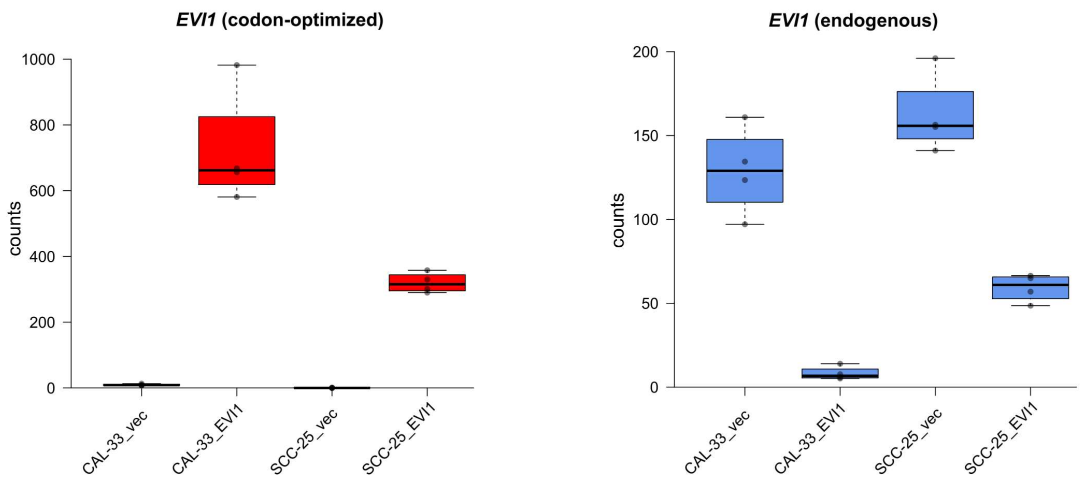
EVI1 (codon-optimized)
1000
800
600
400
200
0
counts
CAL-33_vec
CAL-33_EVI1
SCC-25_vec
SCC-25_EVI1
EVI1 (endogenous)
200
150
100
50
0
counts
CAL-33_vec
CAL-33_EVI1
SCC-25_vec
SCC-25_EVI1

**Figure S7**
